# Climate and Landscape Factors Associated with Buruli Ulcer Incidence in Victoria, Australia

**DOI:** 10.1371/journal.pone.0051074

**Published:** 2012-12-10

**Authors:** Jenni van Ravensway, M. Eric Benbow, Anastasios A. Tsonis, Steven J. Pierce, Lindsay P. Campbell, Janet A. M. Fyfe, John A. Hayman, Paul D. R. Johnson, John R. Wallace, Jiaguo Qi

**Affiliations:** 1 Center for Global Change and Earth Observations, Michigan State University, East Lansing, Michigan, United States of America; 2 Department of Biology, University of Dayton, Dayton, Ohio, United States of America; 3 Department of Mathematical Sciences, University of Wisconsin-Milwaukee, Milwaukee, Wisconsin, United States of America; 4 Center for Statistical Training and Consulting, Michigan State University, East Lansing, Michigan, United States of America; 5 Department of Ecology and Evolutionary Biology, University of Kansas, Lawrence, Kansas, United States of America; 6 Victorian Infectious Diseases Reference Laboratory, North Melbourne, Victoria, Australia; 7 WHO Collaborating Centre for Mycobacterium Ulcerans, Victorian Infectious Diseases Reference Laboratory, North Melbourne, Victoria, Australia; 8 Department of Anatomy and Developmental Biology, Monash University, Melbourne, Australia; 9 Department of Infectious Diseases, Austin Health, Melbourne, Australia; 10 Department of Biology, Millersville University, Millersville, Pennsylvania, United States of America; 11 Department of Geography, Michigan State University, East Lansing, Michigan, United States of America; Universitat Rovira i Virgili, Spain

## Abstract

**Background:**

Buruli ulcer (BU), caused by *Mycobacterium ulcerans (M. ulcerans)*, is a necrotizing skin disease found in more than 30 countries worldwide. BU incidence is highest in West Africa; however, cases have substantially increased in coastal regions of southern Australia over the past 30 years. Although the mode of transmission remains uncertain, the spatial pattern of BU emergence in recent years seems to suggest that there is an environmental niche for *M. ulcerans* and BU prevalence.

**Methodology/Principal Findings:**

Network analysis was applied to BU cases in Victoria, Australia, from 1981–2008. Results revealed a non-random spatio-temporal pattern at the regional scale as well as a stable and efficient BU disease network, indicating that deterministic factors influence the occurrence of this disease. Monthly BU incidence reported by locality was analyzed with landscape and climate data using a multilevel Poisson regression approach. The results suggest the highest BU risk areas occur at low elevations with forested land cover, similar to previous studies of BU risk in West Africa. Additionally, climate conditions as far as 1.5 years in advance appear to impact disease incidence. Warmer and wetter conditions 18–19 months prior to case emergence, followed by a dry period approximately 5 months prior to case emergence seem to favor the occurrence of BU.

**Conclusions/Significance:**

The BU network structure in Victoria, Australia, suggests external environmental factors favor *M. ulcerans* transmission and, therefore, BU incidence. A unique combination of environmental conditions, including land cover type, temperature and a wet-dry sequence, may produce habitat characteristics that support *M. ulcerans* transmission and BU prevalence. These findings imply that future BU research efforts on transmission mechanisms should focus on potential vectors/reservoirs found in those environmental niches. Further, this study is the first to quantitatively estimate environmental lag times associated with BU outbreaks, providing insights for future transmission investigations.

## Introduction

### Buruli Ulcer Disease

Buruli ulcer disease (BU), reported within the top three most frequent human mycobacterial diseases [1], is a rapidly emerging yet neglected tropical disease. BU is endemic in at least 32 tropical and subtropical countries around the world, but most severely impacts areas of sub-Saharan Africa [1], [2]. The disease is caused by infection with the environmental pathogen *Mycobacterium ulcerans* (*M. ulcerans*) and primarily affects the skin, often progressing without pain or fever to the patient [3]. Symptoms can range from a painless, mobile nodule underneath the skin to, if left untreated, large skin ulcerations and sometimes osteomyelitis, a severe bone infection [3]. Treatment with antibiotics can be effective in the pre-ulcerative stage of the disease; however, extensive ulcerations often require surgical removal including amputation of ulcerated limbs [4]. Additional clinical information and comprehensive reviews of BU are available (e.g., [1]).

BU is not transmitted from person-to-person, but rather from direct or indirect contact with *M. ulcerans* in the environment. Studies have frequently linked *M. ulcerans* with aquatic environments (e.g., [5]–[8]); however, very little is known about the ecology of the pathogen and its environmental distribution [1], [9]. To add further difficulties, the human incubation period for BU is unknown, and lag-times between symptom presentation and health-seeking behavior vary on an individual basis, causing the mode(s) of disease transmission to remain unclear.

### Previous BU Studies in Australia

**Figure 1:**
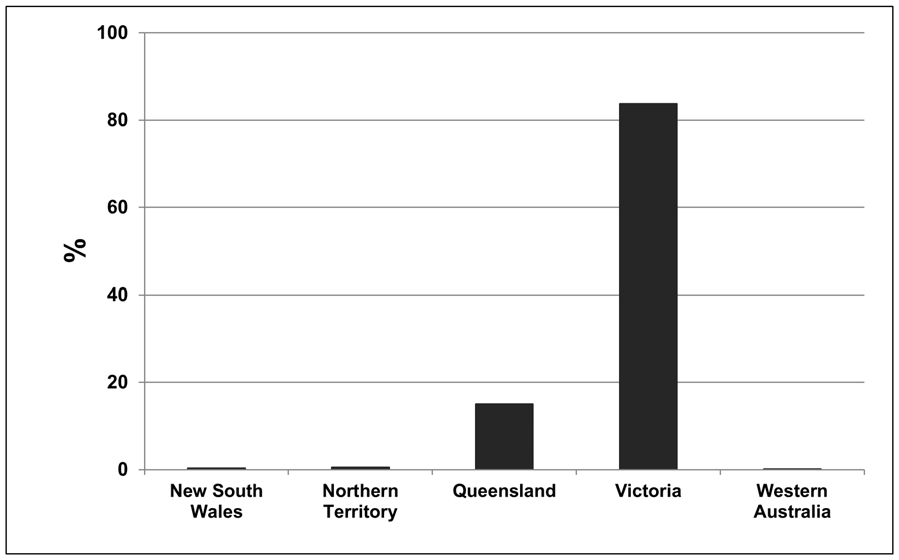
Buruli ulcer incidence in Australia from 1939–2008. The value for each state represents the percentage of total reported Buruli ulcer cases for Australia during this time period.

The highest BU disease incidence rate outside of Africa occurs in Australia, where interestingly, environmental and socio-economic conditions associated with BU patients differ substantially from those in West Africa. The first reported BU case in Australia occurred in 1939 in the Bairnsdale region of Victoria [10]–[13]. Since then, cases have been identified in coastal areas of most Australian states and territories, but about 84% (517 cases through 2008) occurred in Victoria (Figure 1). Unlike the widespread case distribution in some West African countries, outbreaks in Victoria are typically found along the highly populated coast [12], [13]. In both regions; however, BU incidence has increased substantially over the past 20 to 30 years [5], [14], [15]. The most recent and severe Australian BU outbreak to-date occurred in Point Lonsdale, a small coastal town in Victoria, where nearly 70 cases emerged during 2004–2006 (data used in this study).

Several hypotheses on BU transmission in Victoria have emerged in recent years as cases continued to occur and *M. ulcerans* DNA was detected in the environment. Numerous aquatic sources tested positive for *M. ulcerans* DNA including a swamp and an irrigated golf course, the latter of which prompted speculation of pathogen exposure via aerosolization [16], but this was never confirmed. *M. ulcerans* was also detected in aquatic plant biofilm, sediment, and soils [17], [18] leading researchers to focus on possible water contamination sources of *M. ulcerans*, such as landscape disturbance, as part of the BU transmission cycle [2], [10], [13], [16], [19]. Early anecdotal accounts in Victoria lead to the suspicion that flooding may be a precursor to disease outbreaks [10], which has also been speculated in West Africa [20], [21]. Prior to the current study, however, quantitative relationships between climate conditions and BU emergence and/or *M. ulcerans* had yet to be explored.

In recent years, transmission research in Victoria has focused on potential *M. ulcerans* reservoirs and/or BU vectors, in both vertebrate and invertebrate species. A novel study investigating the role of marsupials in the life cycle of *M. ulcerans* in Victoria found evidence to support possums as environmental reservoirs for the pathogen [18]. However, the most widely supported transmission hypothesis in Victoria in recent years is that mosquitoes act as biological or mechanical vectors. Researchers have suggested that mosquito habitats may become contaminated with *M. ulcerans* causing mosquitoes to carry the pathogen internally or externally, thus forming a transmission pathway to humans [12], [22]–[25]. Correlations have been found between BU occurrence and other known mosquito vector-borne diseases in Victoria [22] causing further speculation. In addition, recent studies have: 1) detected *M. ulcerans* DNA in a common species of mosquitoes in Victoria [12], 2) demonstrated that larvae can ingest and maintain the bacteria during early stages of development [3], [24], 3) found pathogen DNA in adult mosquitoes that were infected during the larval stage [25], and 4) demonstrated the successful transfer of the bacteria through three trophic levels with mosquito larvae acting as primary consumers [25]. However, no vector competency studies have been published to date, a necessary criterion for identifying and describing a possible vector [1], [26], [27]. Therefore, it is still unclear whether or not mosquitoes are *M. ulcerans* vectors and if they are, whether the pathogen is carried by the mosquito internally or externally [12].

### Buruli ulcer Research Challenges

Regional climate conditions may play a vital role in BU disease transmission, whether or not *M. ulcerans* is vector-borne, due to the ability of climate events to shift or expand pathogen, reservoir or vector habitats [28]. Annual and seasonal climate change (specifically temperature and precipitation changes), in conjunction with landscape dynamics, may create optimal environmental conditions for disease pathogens and/or vectors to flourish [28]–[30]. Unfortunately, research on climate associations with BU emergence has been lacking due to data availability, quality, and accessibility. Subsequently, this is the first study to investigate both landscape and climate associations that are potentially important to BU ecology, transmission, and risk factors.

In spite of the substantial efforts dedicated to BU research, a number of challenges impose limitations for understanding *M. ulcerans* ecology and BU transmission. For example, most studies have been limited in spatial and/or temporal extent, presenting difficulties in understanding the epidemiology of this disease both regionally and globally. If the occurrence of BU throughout Victoria is entirely random, then localized case studies might be the optimal method for BU research in this region. However, the existence of a large-scale spatio-temporal BU pattern would indicate the presence of external forces driving disease incidence, warranting a regional analysis of potential environmental risk factors for *M. ulcerans* and BU, which until now had not been performed in Victoria.

### Research Questions

The primary purpose of this study was to answer three crucial research questions concerning BU incidence in Victoria:

1. *Is there a statistically significant spatial structure to BU cases indicating the existence of external, regional factors that influence disease emergence?*
2. *Which environmental, i.e., climate and landscape, variables are associated with BU incidence in Victoria at a regional scale?*
3. *Are any of the climate and/or landscape variables linked to BU occurrence consistent with optimal niches of any hypothesized vectors or reservoirs?*

## Materials and Methods

### Study Area

The study area was confined to the southern portion of Victoria, where the majority of Australian BU cases occurred. Although the area is predominantly coastal, the inland extent consists of urban and residential regions surrounded by primarily agricultural land with intermingled forested areas. The region is characterized by a temperate climate with typical maximum summertime (Dec, Jan, Feb) temperatures in the range of 24°C to 27°C and maximum wintertime (Jun, Jul, Aug) temperatures from 12°C to 15°C [31]. The annual rainfall varies substantially with some regions experiencing as little as 500 mm and others as much as 1200 mm [31].

### BU Case Data

Monthly BU incidence data were obtained for Victoria from 1939–2008 from the Department of Health Services, Victoria [10]–[13]. While more than 430 cases were provided in the data set, many had incomplete information (e.g., missing month, year, or location) and thus were removed from this analysis, leaving a total of 318 BU cases. Nearly all of these cases occurred after 1980 (∼95%); therefore, the analysis was limited to data from 1981 to 2008. A total of 302 cases were reported from 90 localities during this time period.

**Figure 2:**
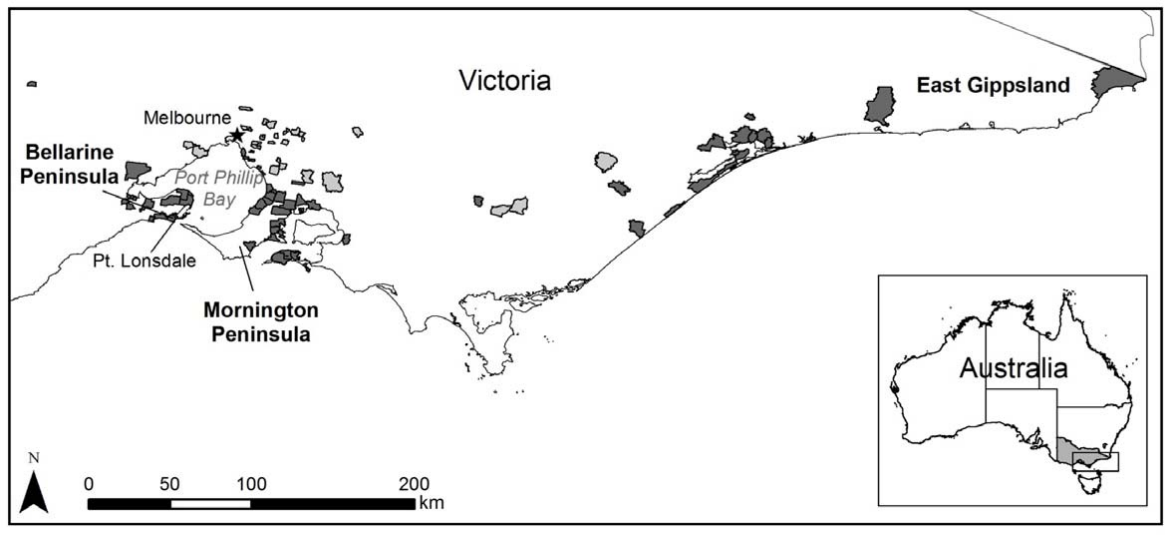
“Endemic” localities in Victoria from 1981–2008. The lighter gray endemic localities may be considered “questionable” as they generally fall outside of the three most frequently identified endemic areas of Bellarine Peninsula, Mornington Peninsula, and East Gippsland.

Data quality and terminology issues were encountered throughout the study and should be noted. The BU research community in Victoria limits the areas considered to be “endemic” to east Gippsland, the Mornington Peninsula, and the Bellarine Peninsula based on conversations with patients in the clinical setting. Despite this, we found that several BU cases (∼10% of the original data) were reported from localities outside of these strictly defined endemic areas (Figure 2). This may be the result of inaccurate patient reporting or insufficient follow-up to determine the likely site of exposure. However, given the uncertainty in the information provided by patients and lack of additional scientific evidence to verify its accuracy, all reported localities were retained for analysis. The resulting errors were assumed to be random, thus having minimum effect on the analysis. In this study, we refer to the localities where BU cases were reported as endemic localities.

Human population data for each endemic locality were obtained from the Australian Bureau of Statistics for the census years of 1981, 1986, 1991, 1996, 2001, and 2006. Because the population density differed among these endemic localities BU cases were standardized to cases per 1000 people for subsequent analyses. The raw number of BU cases from each endemic locality ranged from 1 to 97, while standardized cases ranged from <1 to ∼333.

### Landscape Covariates

To examine potential environmental relationships with BU incidence over a relatively large spatial and temporal scale, commonly used landscape covariates, topography and land use/land cover (LULC), were derived from remotely sensed satellite images. The assumption is that BU outbreaks may be associated with variability in weather patterns, landscape heterogeneity, and land cover types of the local environment.

#### Topography

Topographic characteristics of each locality, including elevation and wetness index, were calculated from a 30 m resolution digital elevation model (DEM) derived from Advanced Spaceborne Thermal Emission and Reflection Radiometer (ASTER) imagery. The wetness index (WI) elucidates the areas of a landscape that are likely to accumulate water when precipitation is present [32]. Zonal statistics, including maximum, minimum, mean and standard deviation, of both elevation and WI were calculated within the boundaries of each endemic locality.

#### Land Use/Land Cover

To analyze the general LULC composition across the study area, Landsat 7 ETM+ satellite imagery (28.5 m spatial resolution) acquired in, or close to, the year 2000 was classified using a hybrid image classification approach first [33] and then further filtered to remove randomly misclassified pixels [34]. The final LULC map contained five unique land cover types, including water, wetland, forest, agriculture, and urban. Due to a lack of ground truth data from the time period the satellite imagery was acquired, land cover types were aggregated into broad, mutually exclusive categories [35] that were more easily confirmed from the Landsat imagery. An accuracy assessment was performed by choosing 50 random “reference” points per cover type [35] and revisiting the original Landsat imagery to determine which type the reference points should belong. An error matrix, generated using the reference points, yielded a kappa statistic [36] of 0.95, indicating that the pixels were 95% more likely to be assigned to the broad class the authors deemed most appropriate than if they had been assigned randomly. From the classified LULC maps, land cover composition (i.e., percentage of each land cover class) within the locality boundaries was derived and used in the subsequent analyses.

### Climate Covariates

Meteorological data were obtained for Victoria from the Australian Bureau of Meteorology (BOM). The data included daily precipitation and maximum and minimum temperatures for the years 1940–2008. Data were removed if they had not been quality checked by the BOM or were indicated as erroneous or suspect. Daily data were converted to monthly averages to match the temporal resolution of the BU case data.

Monthly total and standard deviation of precipitation, as well as averaged maximum and minimum temperatures, were calculated from 1981–2008. Monthly variables were spatially interpolated to climate grids using an Inverse Distance Weighted (IDW) approach [37], [38]. The mean value of pixels that fell within the boundaries of each endemic locality was retained, resulting in a monthly time series of climate observations for all endemic localities. For each locality, the climate data 4 to 24 months prior to the reported BU case date were also included in the statistical analysis to account for unknown lag-times regarding disease transmission, including the time needed for: *M. ulcerans* population increases/decreases in the environment, human exposure to the pathogen (or potential vector), and recognizable symptoms to develop leading patients to engage in health seeking behavior. Because of these factors, combined with minimum reported disease incubation periods of 5–8 weeks [1], climate observations 1 to 3 months prior to reported cases were excluded in this analysis.

### Network Analysis

To address the first objective of this study, the BU disease “network” in Victoria was analyzed to determine whether an underlying spatio-temporal (i.e., structural) pattern of disease incidence occurred at a regional scale. A network consists of two primary components: nodes and links [39]–[41]. Network nodes are defined as the interacting agents, which can be anything. For example, in epidemiological studies of communicable diseases, network nodes typically consist of infected and/or non-infected individuals and are connected (i.e., linked) by contact with one another [42]–[44]. Although network analysis has been fairly limited in disease epidemiology, it is useful for identifying and testing patterns of disease emergence across spatio-temporal scales.

**Figure 3:**
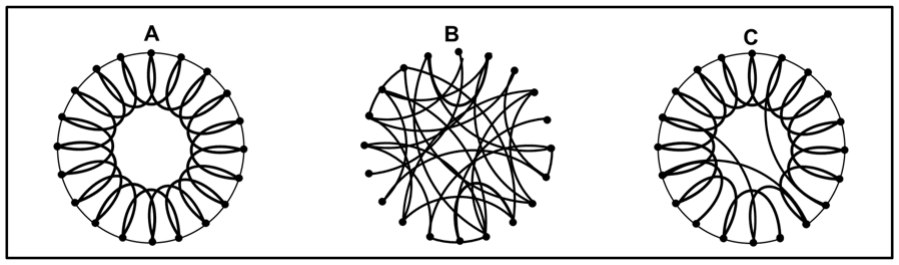
Illustrations of three basic types of networks. Figure (A) shows a regular network, (B) a random network, and (C) a small-world network [40].

There are three basic types of networks: regular, classical random, and small-world. Regular (i.e., ordered) networks contain a fixed number of nodes, each node having the same number of links connecting it in a specific way to a number of neighboring nodes (Figure 3A). A large number of links per node indicates a high local clustering coefficient, *C*. Higher *C* values in regular networks allow for the removal of nodes without breaking the network into non-communicating parts, i.e., a stable network. Classical random networks, on the other hand, consist of nodes that are randomly connected (Figure 3B) and have very small clustering coefficients. As a result, the removal of nodes at random may fracture the network to non-communicating parts, i.e., an unstable network. However, far away nodes can be connected as easily as nearby nodes, allowing information (e.g., diseases, pathogens, reservoirs, or vectors) to be transported across the network much more efficiently than in ordered networks. Most networks in nature are not completely regular or random; rather they tend to be simultaneously stable and efficient in processing information. One such network is the “small-world” network [40], which exhibits a high degree of local clustering, but a small number of long-range connections, making it as efficient in transferring information as random networks (Figure 3C). Another type is the scale-free network (not shown) characterized by the presence of supernodes, where a few nodes are connected to many other nodes and many nodes are connected to only a few nodes, (e.g., an airline route map with a few hubs connected to many locations). Scale-free networks are small-world, thus, are also stable and efficient in transferring information [39].

For BU in Victoria, the disease network was constructed as follows. The nodes consisted of the centroids of BU endemic localities, resulting in *N* = 90 nodes. The node corresponding to the earliest BU case in the study period (1981–2008) was connected to the node where the second BU case occurred. This case was then connected to the next, and so on. However, nodes were not linked to themselves, i.e., if two successive cases in the record occurred at the same location, this link was ignored. This process, which does not affect our results and conclusions, resulted in 216 links (i.e., 216 cases out of the original 302). With this network structure, the localities where BU cases occurred most frequently constitute what are referred to as “supernodes” in the network. “Random” BU networks were constructed as references to assess the characteristics of the actual BU network. First the BU cases from 1981–2008 were chronologically sorted, as with the observed network. Then, each case was assigned a random number between 0.0 and 1.0 and re-sorted based on the value of the random number (from low to high) to obtain a randomized “chronological” sequence. With this dataset, the procedure was repeated 100 times to generate 100 random, or surrogate, BU networks. This approach to constructing random networks preserved the supernodes, but not the links between them and the remaining nodes in the network. When a locality had a high incidence of BU, links would always run through that locality from different nodes (i.e., localities), even when randomizing the chronological order. If the links in the actual network are due to some deterministic force, then these dynamics are destroyed in the random network.

**Figure 4:**
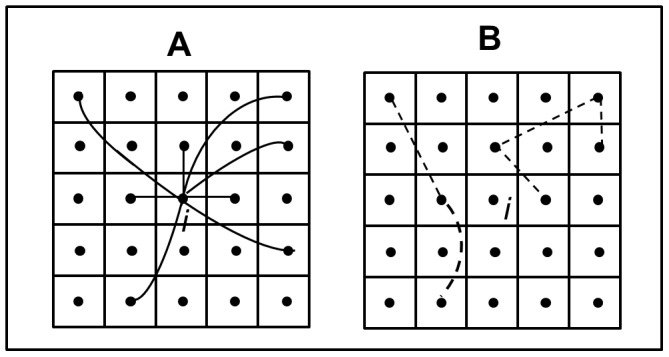
Calculation of the clustering coefficient (*C*). The links between node *i* and other nodes in the network defines the neighborhood of node *i* (A). Node *i* has 8 links, or “neighbors”, denoted as *k_i_*. To calculate the clustering coefficient for node *i* (*C_i_*), we find the number of possible unique connections between the neighbors (*k_i_*) using the formula [*k_i_*(*k_i_*−1)/2], or in this case (8*7)/2 = 28. We then find the number of actual connections (*Δ*_i_) between neighbors, in this case 5 (B). *C_i_* is simply defined as the actual links (*Δ*_i_) divided by the possible links [*k_i_*(*k_i_*−1)/2] in the neighborhood of node *i*. Therefore, *C_i_* = 2*Δ*_i_/(*k_i_*−1)*k_i_* = 0.178 [40].

The clustering coefficients for the Victoria BU network and the random networks were calculated using the procedure illustrated in Figure 4, where first, the clustering coefficient for each node, *C_i_*, is derived. The average *C_i_* over all nodes provides the clustering coefficient of the full network *C*, which can range from 0.0 to 1.0. For a fully connected network, *C* = 1.0.

### Multilevel Regression Modeling

#### Modeling Theory and Approach

To address the second objective a statistical modeling approach was used to find any significant relationships between BU incidence and various climate and landscape conditions across the region and to identify any associated environmental lag-times between habitat conditions and BU occurrence. To accomplish this objective a hierarchical modeling approach was utilized to account for the nested structure of the BU data, as multiple observations of BU cases over time were nested within each endemic locality. Specifically, multilevel Poisson regression models with random effects for endemic localities were developed. The random effect for each locality was introduced to account for autocorrelation in the data from the same locality.

A simple multilevel model can be constructed with Equations 1 and 2 below [45]. The level 1 model (Equation 1) expresses each monthly incidence rate as a function of a locality-specific intercept plus the effects of one time-varying predictor associated with that locality, while the level 2 model in (Equation 2) explains the variation in the locality-specific intercepts as a function of an average intercept across all localities plus the effect of one time-invariant, locality-specific characteristic:
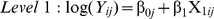
(1)

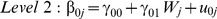
(2)

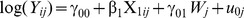
(3)In equation 1 above, ***Y_ij_*** is the monthly incidence rate of BU during month *i* at location *j*, **β_0_**_***j***_ is the random intercept for each location *j* (i.e., endemic locality), **β_1_** is the fixed effect slope for the time-varying predictor **X_1_**_***ij***_. Equation 2 illustrates the components of **β_0_**_***j***_ where **γ_00_** is the grand mean intercept (average of the random intercepts for the different locations), **γ_01_** is the slope for the fixed effect of a time-invariant, location-level predictor called ***W_j_***, and **u_0_**_***j***_ is the estimate of the random effect for each location (i.e., a location-level residual). Equation 3 substitutes the terms from Equation 2 into Equation 1 in place of the **β_0_**_***j***_ term; it therefore represents the combined equation for the multilevel Poisson model.

To develop the models, a data set was constructed such that every month from 1981–2008 had a corresponding number of BU observations for each locality, whether a case was reported or not (months with no cases were assigned a zero). This resulted in a total of 30,240 possible observations (90 localities * 28 years * 12 months). Months with any missing covariates were removed via listwise deletion [46], leaving 27,864 valid observations for model development. The time-varying (i.e., level 1) predictors were the monthly climate covariates, while the time-invariant (i.e., level 2) predictors included elevation, WI, and LULC (Table 1). The models were run in R version 2.10.1 [47] using the *glmer* function from the lme4 package [48], [49].

**Table 1:** Candidate predictor variables for model development.

Predictor Variable	Predictor Level	R
Minimum Elevation	2	−0.54
Maximum Elevation	2	−0.27
Mean Elevation	2	−0.45
Std. Dev. of Elevation	2	−0.16
Minimum Wetness Index	2	0.05
Maximum Wetness Index	2	0.19
Mean Wetness Index	2	0.27
Std. Dev. of Wetness Index	2	0.26
Proportion of Surface Water	2	0.23
Proportion of Wetland	2	0.24
Proportion of Forest	2	0.65
Proportion of Agriculture	2	0.2
Proportion of Urban Area	2	−0.6
Total Monthly Precipitation	1	N/A
Std. Dev. of Monthly Precipitation	1	N/A
Monthly Maximum Temperature	1	N/A
Monthly Minimum Temperature	1	N/A

Std. Dev. = Standard Deviation.

A predictor level of 1 means the variable is time-variant and 2 means the variable is time-invariant. The correlation coefficient (R) between total cases per locality and the level 2 predictor variables is shown in the last column.

Prior to each modeling step, candidate predictor variables were tested for colinearity to ensure that no variables entering a model were correlated with one another. Model performance was evaluated using likelihood ratio tests for nested models [50] and Akaike's Information Criterion (AIC) for non-nested models [51]. The likelihood ratio tests determined whether the addition of new variables to an existing model resulted in a statistically significant improvement (i.e., p≤0.05). For non-nested models, AIC values represent a goodness of fit measure [52] with a lower AIC value indicating a better-fitting model.

#### Model Application

**Figure 5:**
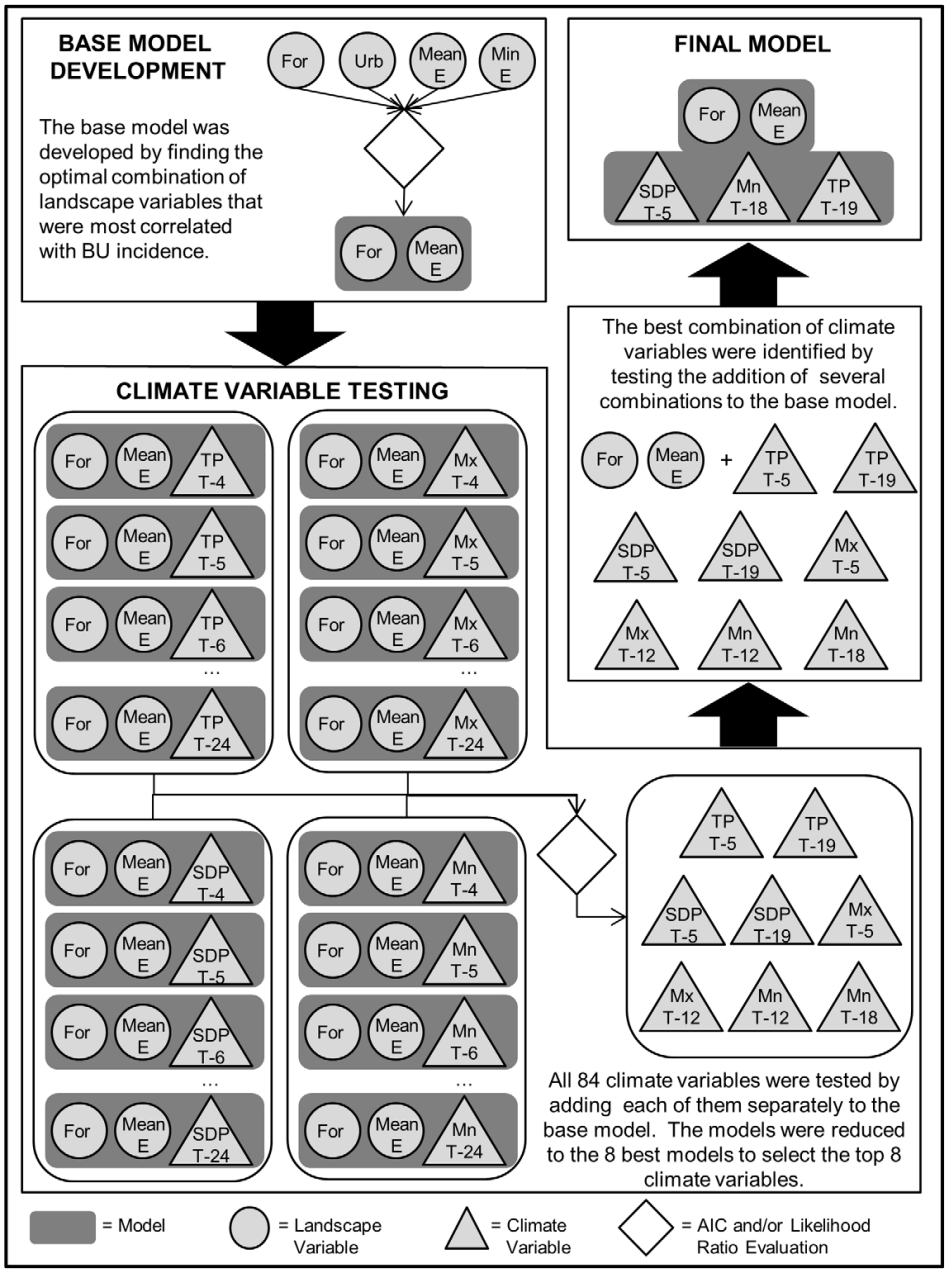
Statistical modeling flowchart. The flowchart shows the multiple stages of model development and variable selection. Variable definitions: “For” = Proportion of Forest, “Urb” = Proportion of Urban, “Min E” = Minimum Elevation, “Mean E” = Mean Elevation, “TP” = Total Precipitation, “SDP” = Standard Deviation of Precipitation, “Mx” = Maximum Temperatures, and “Mn” = Minimum Temperatures. For the climate variables, the notation “T – [number]” refers to the given *variable* at the specified *number* of months prior to BU case incidence.

Models were developed in a multi-step process (Figure 5), without *a priori* assumptions regarding the relationships of the environmental variables and BU prevalence. First, the landscape variables that exhibited the highest correlation with total BU incidence per locality (r>|0.4|) were selected to create a base model (Table 1). Multiple candidate base models were constructed by testing all non-correlated variable combinations. The optimal model was selected by comparing their AIC values. Next, each climate variable was added to the base model and tested separately, resulting in a total of 84 model runs, which were further reduced to eight models by testing model improvement using both likelihood ratio tests and AIC values (Table 2). Different combinations of climate variables from the remaining eight models were added to the base model and examined for the overall best fit.

**Table 2:** The AIC values of models consisting of the base landscape model plus individual climate variables.

Predictor Variable	AIC
Total Monthly Precipitation @ T – 5	581150
Total Monthly Precipitation @ T – 19	586619
Std. Dev. of Monthly Precipitation @ T – 5	565235
Std. Dev. of Monthly Precipitation @ T – 19	622169
Monthly Maximum Temperature @ T – 5	606872
Monthly Maximum Temperature @ T - 12	611374
Monthly Minimum Temperature @ T – 12	622066
Monthly Minimum Temperature @ T – 18	594909

AIC = Akaike's Information Criterion.

Std. Dev. = Standard Deviation.

The base landscape model consisted of Proportion of Forest and Mean Elevation.

The notation “Predictor variable @ T – [number]” refers to the given *variable* at the specified *number* of months prior to BU case incidence.

## Results and Discussion

### Spatial and Temporal Patterns of BU

**Figure 6:**
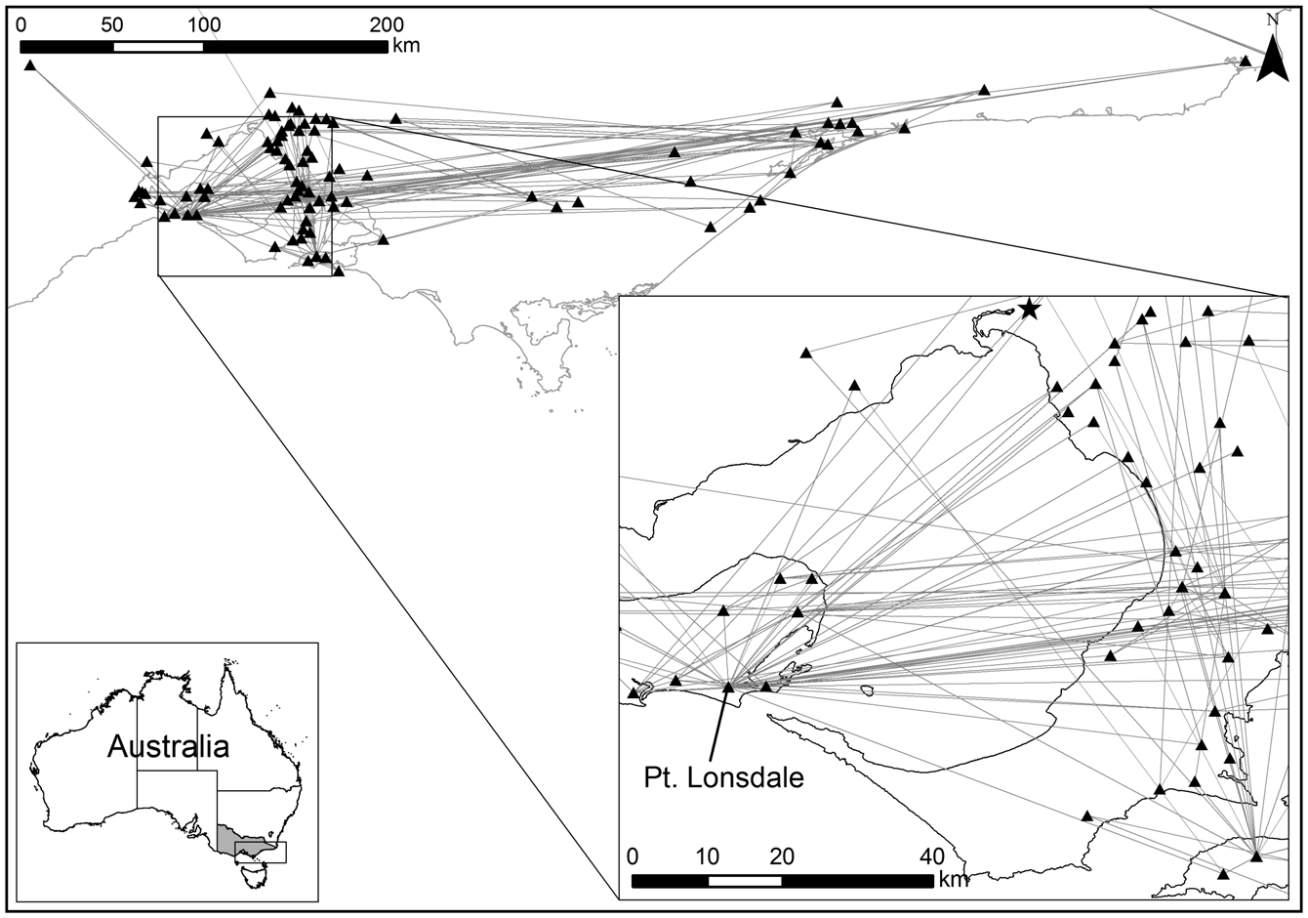
The “actual” Victoria BU disease network from 1981–2008. The centroids of each locality represent the nodes of the network (the black triangles) and the links between consecutive BU cases are represented by lines connecting the nodes.

The Victoria BU network (Figure 6) contains several supernodes, the most notable being the node corresponding to Point Lonsdale. The clustering coefficient of the Victoria network was *C* = 0.32. The same calculation was performed on each of the 100 surrogate networks resulting in a mean *C_r_* of 0.23. Therefore, the null hypothesis ‘*H_o_* = the actual BU network is random’ was rejected at a significance level of 99% (only one random case had a clustering coefficient greater than 0.32). These findings indicate that the BU network structure was not random, i.e., stable, and due to the presence of supernodes, the network was efficient for disease transfer. From an epidemiological perspective, the efficient and stable properties of the BU network in Victoria indicate that BU incidence will not likely diminish on its own and is readily transmitted throughout the region. Additionally, the overall network structure has a greater influence on individual nodes than characteristics or interactions of the nodes themselves [42], suggesting the need to look beyond the local scale aspects of previous BU research. However, the existence of supernodes provides valuable information for developing strategies to control the disease by concentrating treatment and remediation efforts within these localities.

### Environmental Factors Affecting BU Prevalence

The multilevel regression analysis resulted in an overall best-fitting environmental model consisting of a specific combination of optimal land cover, elevation, precipitation, and minimum temperature conditions that are most associated with BU incidence (Table 3). The parameter estimates of this model indicate that localities with a greater proportion of forest cover and lower mean elevations were associated with higher disease incidence. Additionally, climate conditions as early as 19 months in advance may be linked to BU occurrence as results show the total number of cases is positively correlated with precipitation 19 months earlier and with minimum temperature 18 months prior to BU emergence. Both of these variables were highly significant predictors of BU (p<0.0001). Interestingly, lower rainfall variability slightly more than a year after these warm, wet conditions (i.e., 5 months prior to case emergence) also appears to be associated with higher BU risk. However, low rainfall variability could result from very little precipitation throughout the month or, conversely, constant rainfall, which could have very different implications. To clarify this ambiguity, total precipitation five months prior to case emergence was substituted for the standard deviation in the model, which revealed a negative relationship between BU emergence and total rainfall. This suggests that generally dry, rather than wet, conditions may be a more immediate precursor for BU.

**Table 3:** The overall best-fitting environmental Poisson regression model.

Predictor Variable	Estimate	Standard Error	z value	Pr(>|z|)
Intercept	−5.422	0.282	−19.2	<0.001
Proportion of Forest	0.077	0.011	6.9	<0.001
Mean Elevation	−0.015	0.005	−3.0	0.002
Total Monthly Precipitation @ T – 19	0.018	0.000	255.9	<0.001
Std. Dev. of Monthly Precipitation @ T – 5	−0.543	0.003	−172.4	<0.001
Minimum Temperature @ T – 18	0.317	0.002	192.3	<0.001

Std. Dev. = Standard Deviation.

The notation “Predictor variable @ T – [number]” refers to the given *variable* at the specified *number* of months prior to BU case incidence.

Interestingly, the optimal environmental conditions for disease emergence did not include water and wetland variables. However, these results support a recent study in West Africa which found that neither the proportion of water surrounding a village or the proximity to the nearest river were important risk factors for BU [53]. In addition, the significance of forested land cover as a BU risk factor in Victoria is consistent with another West African study showing that villages in Benin surrounded by forested land cover within a 20 km radius tended to have higher BU rates [54]. In addition, living within close proximity to wooded areas and cocoa plantations were identified as risk factors for BU in Cameroon [55]. Further, the importance of topography in high-risk BU habitats in Victoria is also consistent with findings from Benin that showed an increased BU risk in areas with generally lower elevations [54].

### Environmental Niche Implications

The results of this study, particularly the relationships of environmental characteristics with BU incidence, may provide some clues regarding potential vectors or reservoirs for *M. ulcerans* transmission. These environmental conditions may be in favor of certain species, defining an environmental niche that one can use to narrow down certain species as potential vectors. For example, small mammals, especially those that prefer forested habitats, may play a role in BU transmission by acting as a reservoir for *M. ulcerans*, as we found that BU incidence is highly associated with forest cover. This is consistent with the recent discovery of high concentrations of *M. ulcerans* DNA in feces of the common ringtail (Pseudocheirus peregrines) and common brushtail (Trichosurus vulpecula) possum [18], implicating them as potential *M. ulcerans* reservoirs. Ringtail possums forage among tree canopies consuming plant leaves, flowers and fruits, but also include highly nutritive feces as part of their diets (i.e., caecotrophy), while brushtail possums forage on the ground as well as in trees [56], [57]. The semi-arboreal nature of brushtail possums may facilitate their exposure to *M. ulcerans* in the environment. However, the initial source of pathogen exposure for ringtail possums is unclear, even though the persistence of *M. ulcerans* in their intestines may be related to their caecotrophic behavior [18].

It has been speculated that BU transmission is unlikely limited to aquatic ecosystems and may involve several factors, including vegetation, vertebrate hosts and invertebrate vectors in terrestrial habitats as well, suggesting the importance of environmental niche characteristics [18]. In the context of the environmental niche implications, increased rainfall and temperatures could result in an immediate increase in mosquito habitat or population abundance, linking BU cases in the region to flood events in the past. Dry conditions occurring many months after major flood events may result in higher pathogen concentrations in the environment either through the absence of potentially diluting rainfall or by causing potential vectors and/or reservoirs to concentrate within limited areas near accessible water sources. Using possums as an example, this could increase the concentration of potentially contaminated fecal matter within a smaller region, resulting in high enough pathogen levels for disease transmission. There may also be aquatic protozoan or microbial reservoirs that are dependent on initial high precipitation and flooding conditions followed by a dry season [58], [59]. Alternatively, dry conditions could render potential vector or reservoir habitats unsuitable, causing them to migrate to new, potentially non-endemic areas, which could facilitate *M. ulcerans* transmission to traditionally non-endemic regions. Regardless of the potential vectors or reservoirs, the results of this study suggest that BU occurrence is linked to climate variables. With so much still unknown about BU ecology in West Africa, these findings may point future West Africa BU research in this critical, yet previously unexplored direction.

The potential for BU to be a vector-borne disease in Victoria has serious implications for future disease distribution as climate and land use/cover continue to change. Human population in the region grew at an average annual rate of approximately 2.0% from 2005 to 2010 [60], and will likely continue for the foreseeable future. This will likely result in an expansion of anthropogenically disturbed landscapes which may cause ecosystems to shift in favor of *M. ulcerans* and potential vectors/reservoirs. Human modification of landscapes can result in habitat fragmentation and modified hydroperiod conditions [61], [62], which may alter potential vector or reservoir abundance and spatial distribution [2], [54]. These combined affects could create hot-spots for *M. ulcerans* population growth and proliferation, which could then be dispersed throughout non-endemic environments through vector/reservoir behaviors or climatic conditions.

## Conclusions

Despite continued efforts to address data and methodology challenges in BU research, some practical issues remain. The epidemiological data provided by Victoria's Department of Health Services is likely the most spatially and temporally complete BU case data set available; however, many cases were provided without age, sex, and socio-behavioral information. Subsequently, human attributes had to be excluded in this study. Further, the overall number of BU cases was relatively low compared to the range of potential predictor variables. Subsequently, all quality controlled cases within the study period were used for model development, leaving no available case data for validation. However, the overall objective of this research was not to develop a predictive model, but to identify regional environmental risk factors for BU. In addition, the statistical modeling approach accounted for intra-locality autocorrelation, however, additional undiagnosed spatial or spatio-temporal aspects may be present the data, which should be accounted for in future, more in-depth analyses. Finally, epidemiological uncertainties regarding the time elapsed from symptom emergence to medical treatment, which may vary substantially on an individual basis, pose additional challenges in BU research. However, this issue is essentially unavoidable, leaving researchers little choice but to utilize available case data “as-is”. Another major challenge was the lack of environmental data at optimal spatial and/or temporal scales, especially the absence of long-term, systematic *M. ulcerans* sampling within the BU endemic localities. Lack of knowledge regarding environmental pathogen distribution during case emergence promoted speculation on where the bacteria may reside and on human exposure routes, contributing to a level of uncertainty in the modeling results.

This study is among a few that have utilized disease network analysis under circumstances where the vector and/or route of transmission are unknown, demonstrating a novel approach to analyzing BU incidence. The findings from this research confirmed the non-random nature of BU emergence in Victoria at a regional scale, despite local variation, indicating the existence of larger-scale external drivers of disease incidence. Although numerous studies have examined localized behavioral and environmental BU risk factors, this investigation is the first of its kind to identify key landscape and climate features associated with BU incidence in Victoria across a broad scale. These results offer support for the potential interaction of an environmental reservoir for *M. ulcerans* with an invertebrate vector as a conceivable component in the BU transmission cycle. Climate-driven alterations of the spatial distribution of reservoir and/or vector habitats could eventually lead to hot-spots of *M. ulcerans* populations or potentially spread *M. ulcerans* into non-endemic regions, thus representing the efficiency of pathogen transfer offered by the “small-world” BU disease network that was identified. Either circumstance would likely put nearby human populations at risk for *M. ulcerans* exposure. These findings suggest that the BU disease system is likely highly complex, and that this complexity will make it continually difficult to understand and identify key transmission routes due to interacting environmental and ecological components within the extensive climate lag times identified in our models.
